# The Efficacy of Electronic Health Record-Based Artificial Intelligence Models for Early Detection of Pancreatic Cancer: A Systematic Review and Meta-Analysis

**DOI:** 10.3390/cancers18020315

**Published:** 2026-01-20

**Authors:** George G. Makiev, Igor V. Samoylenko, Valeria V. Nazarova, Zahra R. Magomedova, Alexey A. Tryakin, Tigran G. Gevorkyan

**Affiliations:** 1 Predictive Modeling Department, Research Center for Artificial Intelligence in Healthcare, N.N. Blokhin National Medical Research Center of Oncology, Ministry of Health of Russia, 24 Kashirskoe Shosse, Moscow 115522, Russia; 2 Department of Medical Oncology for Gastrointestinal Tumors, N.N. Blokhin National Medical Research Center of Oncology, Ministry of Health of Russia, 24 Kashirskoe Shosse, Moscow 115522, Russia

**Keywords:** artificial intelligence, pancreatic cancer, early detection, electronic health records, machine learning

## Abstract

Background: The persistently low 5-year survival rate for pancreatic cancer (PC) underscores the critical need for early detection. However, population-wide screening remains impractical. Artificial Intelligence (AI) models using electronic health record (EHR) data offer a promising avenue for pre-symptomatic risk stratification. Objective: To systematically review and meta-analyze the performance of AI models for PC prediction based exclusively on structured EHR data. Methods: We systematically searched PubMed, MedRxiv, BioRxiv, and Google Scholar (2010–2025). Inclusion criteria encompassed studies using EHR-derived data (excluding imaging/genomics), applying AI for PC prediction, reporting AUC, and including a non-cancer cohort. Two reviewers independently extracted data. Random-effects meta-analysis was performed for AUC, sensitivity (Se), and specificity (Sp) using R software version 4.5.1. Heterogeneity was assessed using I^2^ statistics and publication bias was evaluated. Results: Of 946 screened records, 19 studies met the inclusion criteria. The pooled AUC across all models was 0.785 (95% CI: 0.759–0.810), indicating good overall discriminatory ability. Neural Network (NN) models demonstrated a statistically significantly higher pooled AUC (0.826) compared to Logistic Regression (LogReg, 0.799), Random Forests (RF, 0.762), and XGBoost (XGB, 0.779) (all *p* < 0.001). In analyses with sufficient data, models like Light Gradient Boosting (LGB) showed superior Se and Sp (99% and 98.7%, respectively) compared to NNs and LogReg, though based on limited studies. Meta-analysis of Se and Sp revealed extreme heterogeneity (I^2^ ≥ 99.9%), and the positive predictive values (PPVs) reported across studies were consistently low (often < 1%), reflecting the challenge of screening a low-prevalence disease. Conclusions: AI models using EHR data show significant promise for early PC detection, with NNs achieving the highest pooled AUC. However, high heterogeneity and typically low PPV highlight the need for standardized methodologies and a targeted risk-stratification approach rather than general population screening. Future prospective validation and integration into clinical decision-support systems are essential.

## Simple Summary

Pancreatic cancer is often detected too late, leading to very low survival rates. Screening everyone is not practical due to the disease’s rarity and high costs. This study explores a new approach: using artificial intelligence (AI) to analyze patients’ existing electronic health records—like doctor’s visit notes and lab results—to identify those at high risk of pancreatic cancer long before symptoms appear. By systematically reviewing existing research, we aimed to determine how accurate these AI tools are. Our findings show they hold significant promise for early detection, which could allow doctors to monitor high-risk patients more closely and ultimately save lives by catching the cancer at a treatable stage. However, challenges such as the potential for false-positive results and the need for further validation in diverse clinical settings must be addressed before its widespread use in clinical practice.

## 1. Introduction

Despite advances in the diagnosis and treatment of malignant neoplasms, the prognosis for pancreatic cancer (PC) remains poor. The five-year survival rate persists at 3–15%, which is largely attributable to the fact that the disease is frequently diagnosed at advanced stages and current systemic treatments have limited effectiveness [1,2]. Although early detection of PC is crucial, the implementation of population-wide screening or early diagnostic programs faces several substantial obstacles [3]. Despite its high mortality, PC is a relatively rare disease. Its low prevalence, combined with the high cost and limited availability of specialized imaging modalities, makes screening of asymptomatic adults impractical [4].

To improve early detection of pancreatic cancer, researchers have increasingly turned to advanced computational approaches for risk assessment and stratification. In recent years, artificial intelligence (AI) has emerged as a powerful tool for the early detection of cancers and other diseases [5,6,7]. In several oncological fields, AI algorithms have demonstrated promising results, not only in improving screening efficacy but also in stratifying the risk of cancer development and progression, thereby identifying high-risk groups [8]. Furthermore, a number of AI-based diagnostic systems have been approved for the automated and non-invasive detection of diseases using medical data in clinical practice [9].

AI algorithms have been applied to the diagnosis of malignancies using clinical and molecular data, as well as via image analysis through radiomics-based techniques [10]. Of particular interest are AI models and algorithms constructed using textual, numerical, and binary variables extracted from electronic medical records (EMRs) [5,11]. This approach is relatively novel and highly feasible for implementation in real-world clinical practice. Unlike methods requiring specialized, non-routine tests or advanced imaging, EMR-based AI models can be seamlessly integrated into existing healthcare systems, offering a scalable and accessible tool for risk stratification and early detection. Nevertheless, the number of studies dedicated to this topic remains limited.

The objective of this study was to describe and comparatively evaluate different artificial intelligence approaches employed for the early diagnosis of pancreatic cancer using data derived from EMRs.

## 2. Methods

### 2.1. Search Strategy

We conducted a comprehensive literature search covering the last fifteen years (2010–2025) across four major online databases: PubMed, MedRxiv, BioRxiv, and Google Scholar. The primary search queries included the following terms: “application of artificial intelligence for pancreatic cancer screening based on database information,” “artificial intelligence and medical records data and pancreatic cancer and screening,” “(‘big data’ OR ‘machine learning’ OR ‘deep learning’ OR ‘artificial intelligence’ OR ‘AI’ OR ‘radiomics’) AND (‘cancer screening’ OR ‘tumor screening’ OR ‘neoplasm screening’ OR ‘oncology screening’ OR ‘early diagnosis’) AND (‘pancreatic cancer’),” “(artificial intelligence for pancreatic cancer screening); (artificial intelligence) AND (cancer screening); (((artificial intelligence) AND (screening)) AND (oncology)) AND (early diagnosis),” “AI-based early detection of pancreatic cancer,” “machine learning in pancreatic cancer screening,” “predictive analytics for pancreatic cancer.”

### 2.2. Inclusion and Exclusion Criteria

Studies were eligible for inclusion if they met the following criteria: utilization of EMR-derived data exclusively, without incorporating other data modalities (e.g., radiological imaging, molecular profiling); application of artificial intelligence methods as the primary analytical approach; availability of sufficient outcome data to enable performance assessment—specifically, AUC/AUROC metrics; a study objective focused on early detection of pancreatic cancer, which required inclusion of a population without pre-existing pancreatic cancer. Studies were excluded if they met any of the following criteria: published only as letters or abstracts; lacked quantitative performance metrics, used AI only for data extraction rather than prediction, included only patients with confirmed cancer, without a non-cancer comparison group. We followed the Preferred Reporting Items for Systematic Reviews and Meta-Analyses (PRISMA) guidelines [12]. This study was registered in PROSPERO, registration number is CRD420261280741.

### 2.3. Data Extraction

Two researchers independently screened the literature. The following information was extracted from each eligible study: country of origin; data source, including the specific databases and repositories used; type and specific architecture of the AI models; validation methods employed; target outcomes and study design; study population characteristics; type of data inputs; number of patients with pancreatic cancer; total sample size. Performance metrics extracted included: sensitivity (Se), specificity (Sp), AUROC/C-index, NPV (negative predictive value), and PPV (positive predictive value). Additionally, bibliographies of relevant reviews, articles, and monographs were screened for supplemental references. Any discrepancies were resolved through discussion and consensus.

### 2.4. Statistical Analysis and Quality Appraisal

Descriptive and qualitative comparisons of the extracted data were used to summarize differences across AI approaches and methodological frameworks. Meta-analysis and data visualization were conducted using R version 4.5.1 (R Foundation for Statistical Computing), employing the metafor package (version 4.8-0). Random-effects models were applied to obtain pooled estimates of predictive performance and 95% prediction intervals. Fixed-effects models were used for meta-regression analyses and for obtaining pooled estimates within predefined algorithm categories. Heterogeneity across included studies was assessed using Cochran’s Q statistic and the I^2^ index. For meta-analyses including five or more studies, publication bias was evaluated using funnel plots and Egger’s regression test. Given the anticipated high heterogeneity in sensitivity and specificity estimates, we initially employed univariate random-effects meta-analysis. However, we acknowledge that bivariate models (such as HSROC (Hierarchical Summary Receiver Operating Characteristic)) are more appropriate for diagnostic accuracy meta-analyses and recommend their use in future studies when sufficient data are available.

The methodological quality of the studies included in the systematic review was assessed using the Newcastle–Ottawa Scale [13]. The authors evaluated each study by applying the Newcastle–Ottawa Scale criteria across three domains: selection of participants, comparability of groups, and outcome ascertainment. A point was awarded for each criterion that was fully met, while no points were given for unmet or partially met criteria. In accordance with the Agency for Healthcare Research and Quality guidelines, the overall quality of each study was then translated into a rating of “poor”, “fair”, or “good” based on the total score.

## 3. Results

### 3.1. Characteristics of the Included Studies

**Figure 1:**
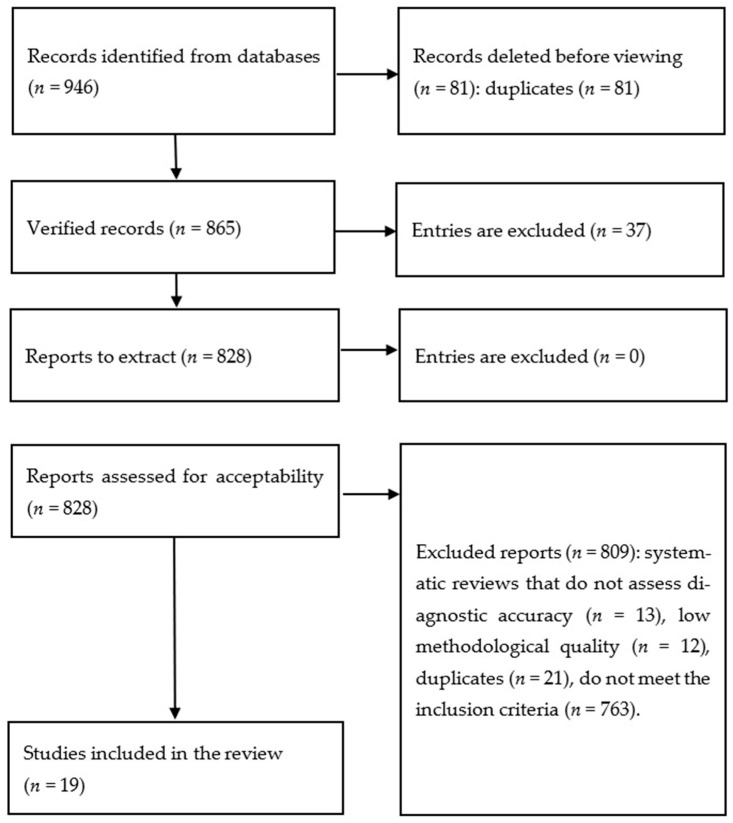
Study selection methodology.

A total of 946 records were identified through database searches. The study selection process is depicted in Figure 1, and detailed characteristics of the studies included in the analysis are presented in Table 1. Nineteen studies met the predefined inclusion criteria. Most of the included studies were retrospective cohort analyses employing internal validation strategies, such as train–test split of the available datasets. Across all studies, the primary outcome measure was consistent—either the diagnosis of pancreatic cancer or the time to diagnosis (Table 1). All studies relied exclusively on EMR-derived data, which included clinical documentation related to healthcare encounters; in several cases, socio-demographic survey data were also incorporated.

For a clearer understanding of the role and future potential of AI in early cancer detection, it is important to distinguish between two related terms—machine learning (ML) and deep learning (DL). Machine learning refers to a broad class of algorithms capable of learning from data and improving their predictive performance over time. Deep learning, by contrast, is a specialized subset of ML that utilizes complex multilayer neural networks capable of capturing intricate nonlinear relationships [14]. For clarity, Table 1 lists ML and DL as separate AI categories, although DL is technically a subset of ML. Across the included studies, a variety of AI algorithms were used, including XGBoost (XGB), Random Forests (RFs), logistic regression (LogReg), ensemble models, neural networks (NNs), and Light Gradient Boosting (LGB).

**Table 1:** Summary of studies on AI-based early detection of pancreatic cancer using EMR data.

Reference	AI Type	AI Models	Outcome Measures	Population Characteristics	PC Cases (*n*)	Total Sample (*n*)	Model Performance Metrics
Chen, Q. et al. [15]	ML	XGBoost	Diagnosis of PC; ability to distinguish early-stage PC patients from matched controls	Early-stage PC defined as surgically managed cases; controls matched by region and observation period	3322	53,152	Se = 60%, Sp = 89.8%, PPV = 0.07%, AUC = 0.84 (95% CI: 0.83–0.85)
Chen, W., Zhou, Y., Xie, F. et al. [16]	ML	Random Forest	Detection of PC or PC-related death within 18 months	Adults aged 50–84 years with ≥1 clinical visit during 2008–2017; continuous enrollment ≥ 12 months prior to index date	1792	1,801,931	Se = 56.6%, Sp = 79.6%, PPV = 1.1%, C-index = 0.77
Matchaba, S. et al. [17]	ML	Ensemble Model	PC diagnosis predicted 1–2 years prior to clinical diagnosis	Patients with ≥3 healthcare interactions	8438	18,987	Se = 85.61%, Sp = 76.18%, PPV = 1.1%, AUC = 0.89
Appelbaum, L. et al. [11]	ML	Logistic Regression, Neural Network	PC diagnosis predicted 180, 270, and 365 days prior to clinical diagnosis	Patients with ≥6 months of observation prior to diagnosis (cases) or prior to last visit (controls)	594	101,381	AUROC = 0.71 (95% CI: 0.67–0.76), PPV = 0.93%
Park, J. et al. [18]	DL	LogReg, NN, Random Masking, XGB, Black-box model	PC diagnosis predicted within 12 months	Patients with ≥1 documented risk factor (smoking, obesity, diabetes, chronic pancreatitis); exclusion of those undergoing treatment for chronic pancreatitis	834	9057	AUROC: LogReg 0.491; XGB 0.501; Black-box 0.644; NN (no masking) 0.649; NN + Random Masking 0.671
Chen, W., Butler, R. et al. [19]	ML	Random Forest	PC diagnosis or death from PC within 3 years after elevated HbA1c	Adults aged 50–84 years with ≥1 elevated HbA1c; exclusion of diabetes patients	319	109,266	Se = 60%, Sp = 80.3%, PPV = 2.5%, AUROC = 0.812
Malhotra, A. et al. [5]	ML	LogReg, RF	Binary classification: PC case vs. control with unrelated cancer	Patients aged 15–99 with primary PC; controls with other cancers	1139	5695	Se = 65%, Sp = 57%, AUC = 61%, PPV = 32.5%
Hsieh, M. et al. [20]	ML	LogReg, NN	PC diagnosis among patients with type 2 diabetes	Adults > 20 years with newly diagnosed type 2 diabetes; exclusion of those with prior PC	3092	1,358,634	PPV: LogReg 0.995, NN 0.996; AUROC: LogReg 0.727, NN 0.605
Muhammad, W. et al. [21]	DL	Neural Network	Binary: PC diagnosis within 4 years of survey	General population; PC diagnosed < 4 years before survey	898	800,114	Se = 87.3%, Sp = 80.8%, AUROC = 0.86, NPV = 99.997%, PPV = 0.1%
Placido, D. et al. [22]	DL	Transformer	Risk of PC at 3, 6, 12, 36, and 60 months	General population; archival EMRs	26,403	8,123,446	AUC = 0.879, PPV = 0.32%
Xiaodong, Li et al. [23]	ML, DL	XGB + Deep NN	PC diagnosis within a 24-month prediction window	Adults ≥ 35 years seeking medical care	4361	265,225	Se = 54.35%, AUROC = 0.809, PPV = 67.62%
Zhao, D. et al. [24]	ML	Weighted Bayesian Network Inference	Binary: PC vs. non-PC	Randomly selected controls plus symptomatic non-PC patients	98	15,069	Se = 84.7%, Sp = 85.2%, AUROC = 0.910
Chen, W., Zhou, B., Jeon, C. et al. [25]	ML	RF, XGB	Time-to-event: PC diagnosis or PC-related death within 18 months	Adults aged 50–84, ≥1 clinical visit, no prior PC	1792	1,800,000	RF: AUC 0.767; XGB: AUC 0.779; PPV ≈1%
Jia, K. et al. [26]	ML	PrismNN, LogReg	PC risk prediction 6–18 months after index date	Adults > 40 with ≥16 records over 2 years	35,387	1,535,468	PrismNN: Se = 35.9%, Sp = 95.3%, AUROC = 0.826; LogReg: AUROC = 0.800
Cichosz, S. et al. [27]	ML	Random Forest	PC development within 3 years after diabetes onset	Adults > 50 with new-onset diabetes	716	1432	AUROC = 0.74; Se 21.4% at high specificity; NPV up to 99.8%
Shih-Min Chen et al. [28]	ML	Linear Discriminant Analysis	PC diagnosis within 4 years post anti-diabetes therapy	Type 2 diabetes patients ≥ 40 years	89	66,384	Se 86.11%, Sp 84.03%, AUROC 0.907, PPV 0.02%
Zhichao Yang et al. [29]	DL	NN, Transformer	Disease-/Outcome-agnostic prediction: all ICD oncology codes at next visit	General healthcare-seeking population; PC subgroup ≥ 45 years without other cancers	4639	6,475,218	AUROC = 0.82, PPV = 8.8%
Zhu, W. et al. [30]	ML	Elastic-Net Regularized LogReg	PC diagnosis within 2.5–3 years	Adults with ≥3 years of continuous EMR data	1932	53,741	At 1st percentile threshold: Se = 6.78%, Sp = 99.01%, NPV = 99.93%, PPV = 0.65%, AUROC = 0.742
Akmeşe, Ö. et al. [31]	ML	LGBM, Bagging, CatBoost and others	Binary: PC vs. non-PC	PC-patients, non-cancerous (benign) pancreatic/hepatobiliary disease patients, healthy controls. In addition to clinical data, laboratory data were also used	199	590	LGBM (Best Model): accuracy = 98.8%, precision = 99%, recall = 99%, F1-Score = 0.99, Se = 99%, Sp = 98.7%

### 3.2. Quality Assessment

The methodological quality of the included studies, assessed using the Newcastle–Ottawa Scale, was variable but generally acceptable for observational research (Table 2). The majority of the studies demonstrated an adequate design regarding participant selection and outcome assessment, receiving a rating of “good” or “fair” in terms of Methodological Quality. However, some heterogeneity was noted, particularly in the comparability of study groups and control for confounding factors.

**Table 2:** Study quality appraisal outcomes using the Newcastle–Ottawa Scale.

Study	Selection (/4)	Comparability (/2)	Outcome (/3)	Methodological Quality
Chen, Q. et al. [15]	4	0	2	6 (Fair)
Chen, W., Zhou, Y., Xie, F. et al. [16]	4	1	2	7 (Fair)
Matchaba, S. et al. [17]	4	0	2	6 (Fair)
Appelbaum, L. et al. [11]	4	1	2	7 (Fair)
Park, J. et al. [18]	4	2	3	9 (Good)
Chen, W., Butler, R. et al. [19]	4	2	3	9 (Good)
Malhotra, A. et al. [5]	4	2	3	9 (Good)
Hsieh, M. et al. [20]	4	2	3	9 (Good)
Muhammad, W. et al. [21]	4	2	2	8 (Good)
Placido, D. et al. [22]	4	2	2	8 (Good)
Xiaodong Li et al. [23]	3	0	2	5 (Poor)
Zhao, D. et al. [24]	4	2	3	9 (Good)
Chen, W., Zhou, B., Jeon, C. et al. [25]	4	2	2	8 (Good)
Jia, K. et al. [26]	4	2	2	8 (Good)
Cichosz, S. et al. [27]	4	1	3	8 (Good)
Shih-Min Chen et al. [28]	4	2	2	8 (Good)
Zhichao Yang et al. [29]	4	2	2	8 (Good)
Zhu, W. et al. [30]	3	1	3	7 (Fair)
Akmeşe, Ö. et al. [31]	3	0	2	5 (Poor)

### 3.3. Meta-Analytic Assessment of AUC

**Figure 2:**
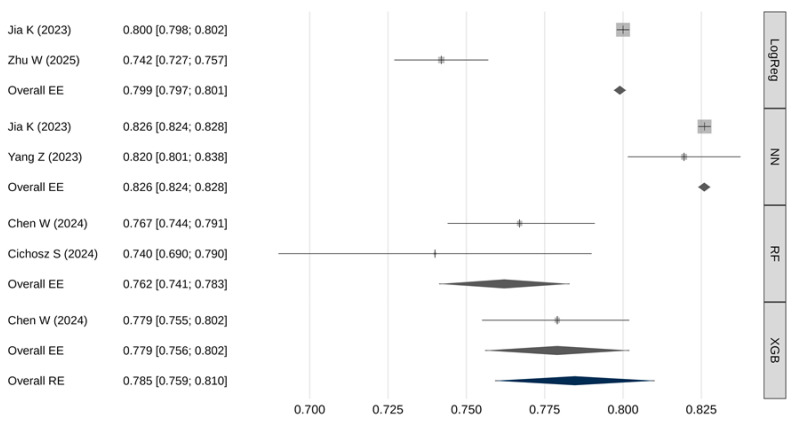
Meta-analysis results for the AUC [25,26,27,29,30].

Not all studies provided adequate data for inclusion in the AUC meta-analysis. Table 3 and Figure 2 summarize the pooled AUC results. The overall pooled AUC using a random-effects model was 0.785 [95% CI: 0.759–0.810], with a 95% prediction interval ranging from 0.716 to 0.853. There was substantial heterogeneity across studies (I^2^ = 99.5%, Q(df) = 458.6, *p* < 0.001). No strong evidence of publication bias was identified (β = 0.82; [95% CI: 0.80–0.84]; *p* = 0.406).

Meta-regression demonstrated a statistically significant association between model type and AUC values (Q(3) = 398, *p* < 0.001). Pairwise comparisons using fixed-effects models showed (Table 4): neural networks had significantly higher AUROC values compared to logistic regression, RF, and XGB models (*p* < 0.001); logistic regression models had significantly higher AUROC than RF models (*p* < 0.001).

### 3.4. Meta-Analytic Assessment of Sensitivity

**Figure 3:**
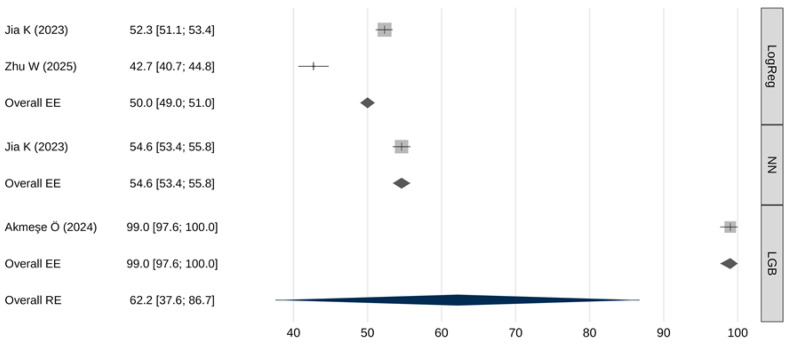
Meta-analysis results for sensitivity [26,30,31].

Table 5 and Figure 3 summarize the pooled sensitivity results. The overall pooled sensitivity (random effects) was 62.2% [95% CI: 37.6–86.7], with a 95% prediction interval from 7.2% to 100%. Extremely high heterogeneity was observed (I^2^ = 99.9%, Q(df) = 3636, *p* < 0.001). No significant publication bias was identified (β = 0.65; [95% CI: 2.43–3.73]; *p* = 0.981).

Meta-regression indicated that sensitivity differed significantly across AI model types (Q(2) = 3569, *p* < 0.001). Table 6 presents the results of pairwise comparisons of meta-analytic sensitivity estimates obtained using the fixed-effects models. LGB models demonstrated the highest sensitivity—statistically superior to both neural networks and logistic regression (*p* < 0.001), neural networks also demonstrated a slightly higher sensitivity compared to logistic regression (*p* < 0.001).

### 3.5. Meta-Analytic Assessment of Specificity

**Figure 4:**
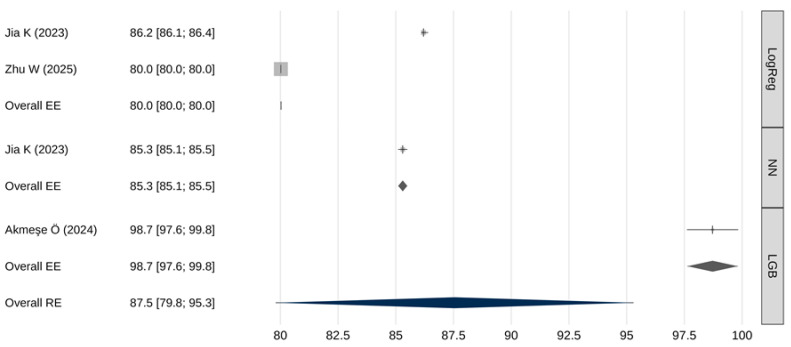
Meta-analysis results for specificity [26,30,31].

A meta-analysis of the specificity of AI models was also performed (Table 7 and Figure 4). The overall pooled specificity (random effects) was 87.5% [95% CI: 79.8–95.3], with a 95% prediction interval from 70.2% to 100%. Again, extremely high heterogeneity was present (I^2^ = 100%, Q(df) = 10,695, *p* < 0.001), and there was a trend toward publication bias (β = 0.8; [95% CI: 0.8–0.8;] *p* = 0.062).

The meta-analytic regression model revealed a statistically significant association between the type of predictive model and specificity estimates (Q(2) = 3913, *p* < 0.001). Table 8 presents the results of pairwise comparisons of meta-analytic specificity estimates obtained using fixed-effects models. LGB models demonstrated a statistically significantly higher specificity compared to both logistic regression models and neural network-based models (*p* < 0.001). Neural networks also showed a somewhat higher specificity than logistic regression (*p* < 0.001).

## 4. Discussion

This systematic review and meta-analysis demonstrate the substantial potential of artificial intelligence models trained on electronic medical record (EMR) data to address one of the most critical challenges in oncology—the early detection of pancreatic cancer (PC). The pooled AUC estimate of 0.785 [95% CI: 0.759–0.810] indicates overall good and clinically meaningful discriminatory performance of AI-based models. A notable finding of this review is the statistically significant difference in performance across AI model types. Neural network-based models consistently demonstrated the highest AUROC values, likely reflecting their ability to capture complex nonlinear relationships and temporal patterns within high-dimensional EMR data—patterns that may be undetectable by more traditional models such as logistic regression. However, while neural networks achieved the highest pooled AUC, caution is warranted in interpreting this result. Performance disparities could be influenced by study-specific factors—including sample size, feature engineering, temporal modeling, and validation design—rather than algorithmic advantage alone.

Performance comparisons across key metrics revealed additional important distinctions. In terms of sensitivity, LGB models markedly outperformed both neural networks and logistic regression (99% [97.6–100] vs. 54.6% [53.4–55.8] and 50% [49–51], respectively). Such high sensitivity makes LGB models particularly attractive for screening applications where failure to detect disease carries significant clinical risk. However, this exceptionally high sensitivity was reported in a single study, warranting cautious interpretation and the need for external validation. Similarly, LGB models demonstrated significantly higher specificity than neural networks or logistic regression (98.7% [97.6–99.8] vs. 85.3% [85.1–85.5] and 80% [80–80], respectively). Neural networks, meanwhile, exhibited intermediate—yet significantly superior—performance compared with logistic regression across both sensitivity and specificity metrics.

Despite encouraging results, the analysis revealed extremely high heterogeneity in sensitivity and specificity estimates between studies (I^2^ ≥ 99.9%). This could be explained by substantial differences in study design, variations in the studied populations, and the types of predictors used. Our meta-analysis is limited by the high methodological heterogeneity of the included works: they differed in design (case–control vs. cohort), outcomes (binary classification vs. time-to-event), time horizons (3–60 months), population composition (general population and at-risk cohorts), as well as classification thresholds defining sensitivity and specificity. Some studies used enriched cohorts or external data sources, which reduces comparability with models trained on ‘pure’ EMRs of the general population. Under such conditions, aggregating Se/Sp in univariate models may yield biased estimates; due to the high heterogeneity, the results for Se and Sp should be interpreted with caution. Therefore, in future analyses, once a larger number of studies on this topic become available, alternative approaches such as the bivariate HSROC model should be employed for a more accurate estimation.

Differences in prediction windows and outcome definitions are important sources of heterogeneity, but a stratified meta-analysis was not possible due to incomplete reporting. Few studies provided performance metrics for specific time horizons, and the number of comparable studies within each stratum was too low to allow robust subgroup analysis. This limitation underscores the need for standardized reporting of time-stratified results in future research on AI-based pancreatic cancer prediction.

The extremely low PPVs observed in most studies underscore a fundamental challenge in pancreatic cancer screening, driven by the disease’s low prevalence. Even a model with high specificity will generate a large number of false-positive results when applied to the general population, potentially leading to unnecessary diagnostic procedures. Consequently, an AI implementation strategy should focus not on universal screening, but on risk stratification and the identification of high-risk patient groups. Within these high-risk cohorts, more in-depth analyses using AI models should be conducted to define subgroups for targeted instrumental examination.

Our analysis highlights the critical need for AI methodologies that can effectively manage the inherent complexity, non-linearity, and temporal dynamics of longitudinal EHR-data. This imperative is echoed by contemporary methodological innovations in modeling other complex, chronic conditions. The DEPOT framework, for example, utilizes a graph-based AI approach specifically engineered to address the challenges of fragmented and heterogeneous EHR data [32]. By constructing an age-similarity graph and employing representation learning, DEPOT successfully identified distinct, high-risk progression trajectories for chronic kidney disease within a diabetic cohort. Importantly, this model’s ability to isolate a high-risk trajectory enriched with specific cancer phenotypes powerfully illustrates the principle of targeted risk stratification [33]. This aligns with our central conclusion that the early detection of pancreatic cancer will be most feasible and effective when AI models are applied to identify and monitor enriched high-risk subpopulations, rather than through untargeted general screening.

## 5. Conclusions

Artificial intelligence models trained on electronic medical record data represent a promising tool for improving the early detection of pancreatic cancer, demonstrating good discriminatory ability in certain studies. However, high heterogeneity, typically low positive predictive value, and the absence of further validation currently preclude their use as stand-alone screening tools. Their effective clinical implementation relies not on replacing clinicians, but on integrating AI systems as decision-support tools capable of identifying patients at elevated risk who may benefit from closer evaluation or targeted diagnostic testing.

Successful implementation of these technologies will require further prospective research, methodological standardization, and improved interpretability of AI models, as well as seamless integration into clinical workflows. By addressing these challenges, AI-driven risk stratification has the potential to transform the diagnostic landscape for pancreatic cancer and mitigate delays in detection—ultimately improving patient outcomes.

## Figures and Tables

**Table 3:** Meta-analysis results for the AUC.

Model Type	Study	AUC	95% CI	Heterogeneity Statistics			
				I^2^	Q	df	*p*
LogReg	Jia, K. (2023) [26]	0.8	0.798; 0.802	%			
LogReg	Zhu, W. (2025) [30]	0.742	0.727; 0.757	%			
LogReg	Overall EE	0.799	0.797; 0.801	98.3%	58.8	1	<0.001
NN	Jia, K. (2023) [26]	0.826	0.824; 0.828	%			
NN	Yang, Z. (2023) [29]	0.82	0.801; 0.838	%			
NN	Overall EE	0.826	0.824; 0.828	0%	0.515	1	0.473
RF	Chen, W. (2024) [25]	0.767	0.744; 0.791	%			
RF	Cichosz, S. (2024) [27]	0.74	0.69; 0.79	%			
RF	Overall EE	0.762	0.741; 0.783	0%	0.955	1	0.328
XGB	Chen, W. (2024) [25]	0.779	0.755; 0.802	%			
XGB	Overall EE	0.779	0.756; 0.802	0%	0	0	>0.999
	Overall RE	0.785	0.759; 0.81	%			

**Table 4:** Pairwise comparisons of meta-analytic AUC estimates.

Comparison	Difference in AUC	95% CI	*p*
NN vs. LogReg	0.027	0.024; 0.03	**<0.001**
RF vs. LogReg	−0.037	−0.058; −0.016	**<0.001**
XGB vs. LogReg	−0.02	−0.043; 0.003	0.09
RF vs. NN	−0.064	−0.085; −0.043	**<0.001**
XGB vs. NN	−0.047	−0.07; −0.024	**<0.001**
XGB vs. RF	0.017	−0.014; 0.048	0.287

**Table 5:** Meta-analysis results for sensitivity.

Model Type	Study	Sensitivity (Se)	95% CI	Heterogeneity Statistics			
				I^2^	Q	df	*p*
LogReg	Jia, K. (2023) [26]	52.3	51.1; 53.4	%			
LogReg	Zhu, W. (2025) [30]	42.7	40.7; 44.8	%			
LogReg	Overall EE	50	49; 51	98.5%	66.6	1	<0.001
NN	Jia, K. (2023) [26]	54.6	53.4; 55.8	%			
NN	Overall EE	54.6	53.4; 55.8	0%	0	0	>0.999
LGB	Akmeşe, Ö. (2024) [31]	99	97.6; 100	%			
LGB	Overall EE	99	97.6; 100	0%	0	0	>0.999
	Overall RE	62.2	37.6; 86.7	%			

**Table 6:** Pairwise comparisons of meta-analytic sensitivity estimates.

Comparison	Difference i95.	95% CI	*p*
NN vs. LogReg	4.6	3.1; 6.1	<0.001
LGB vs. LogReg	49	47.3; 50.7	<0.001
LGB vs. NN	44.4	42.6; 46.2	<0.001

**Table 7:** Meta-analysis results for specificity.

Model Type	Study	Specificity (Sp)	95% CI	Heterogeneity Statistics			
				I^2^	Q	df	*p*
LogReg	Jia, K. (2023) [26]	86.2	86.1; 86.4	%			
LogReg	Zhu, W. (2025) [30]	80	80; 80	%			
LogReg	Overall EE	80	80; 80	100%	6782	1	<0.001
NN	Jia, K. (2023) [26]	85.3	85.1; 85.5	%			
NN	Overall EE	85.3	85.1; 85.5	0%	0	0	>0.999
LGB	Akmeşe, Ö. (2024) [31]	98.7	97.6; 99.8	%			
LGB	Overall EE	98.7	97.6; 99.8	0%	0	0	>0.999
	Overall RE	87.5	79.8; 95.3	%			

**Table 8:** Pairwise comparisons of meta-analytic specificity estimates.

Comparison	Difference in Specificity (Sp)	95% CI	*p*
NN vs. LogReg	5.3	5.1; 5.5	<0.001
LGB vs. LogReg	18.7	17.6; 19.8	<0.001
LGB vs. NN	13.4	12.3; 14.5	<0.001

## Data Availability

The original contributions presented in this study are included in the article. Further inquiries can be directed to the corresponding author.

## Abbreviations

The following abbreviations are used in this manuscript:

PC	Pancreatic cancer
AI	Artificial Intelligence
EHR	Electronic health record
Se	Sensitivity
Sp	Specificity
LogReg	Logistic Regression
LGB	Light Gradient Boosting
RF	Random Forests
NN	Neural Network
XGB	XGBoost
CI	Confidence interval
ML	Machine learning
DL	Deep learning
AUC	Area under the curve
AUROC	Area under the receiver operating characteristic curve
